# Inconsistencies in Modeling the Efficacy of the Oncolytic Virus HSV1716 Reveal Potential Predictive Biomarkers for Tolerability

**DOI:** 10.3389/fmolb.2022.889395

**Published:** 2022-06-15

**Authors:** Faith Howard, Joe Conner, Sarah Danson, Munitta Muthana

**Affiliations:** ^1^ Department of Oncology and Metabolism, University of Sheffield, Sheffield, United Kingdom; ^2^ Virtuu Biologics/Sorrento Therapeutics, Biocity Scotland, Newhouse, United Kingdom; ^3^ Sheffield Experimental Cancer Medicine Centre and Weston Park Cancer Centre, Weston Park Hospital, University of Sheffield, Sheffield, United Kingdom

**Keywords:** oncolytic virotherapy, preclinical modeling, T helper cells, tolerability, biomarker

## Abstract

Treatment with HSV1716 *via* intralesional administration has proven successful for melanoma patients with the hope that oncolytic virotherapy would become another weapon in the systemic anticancer therapy (SACT) arsenal. In addition to challenges surrounding the systemic delivery of oncolytic viruses (OVs), problems associated with its *in vivo* modeling have resulted in low predictive power, contributing to the observed disappointing clinical efficacy. As OV’s efficacy is elicited through interaction with the immune system, syngeneic orthotopic mouse models offer the opportunity to study these with high reproducibility and at a lower cost; however, inbred animals display specific immune characteristics which may confound results. The systemic delivery of HSV1716 was, therefore, assessed in multiple murine models of breast cancer. Tolerability to the virus was strain-dependent with C57/Bl6, the most tolerant and Balb/c experiencing lethal side effects, when delivered intravenously. Maximum tolerated doses were not enough to demonstrate efficacy against tumor growth rates or survival of Balb/c and FVB mouse models; therefore; the most susceptible strain (Balb/c mice) was treated with immunomodulators prior to virus administration in an attempt to reduce side effects. These studies demonstrate the number of variables to consider when modeling the efficacy of OVs and the complexities involved in their interpretation for translational purposes. By reporting these observations, we have potentially revealed a role for T-cell helper polarization in viral tolerability. Importantly, these findings were translated to human studies, whereby a Th1 cytokine profile was expressed in pleural effusions of patients that responded to HSV1716 treatment for malignant pleural mesothelioma with minimal side effects, warranting further investigation as a biomarker for predictive response.

## Introduction

The advent of immunotherapies combined with or without chemotherapy has become an alternative first-line or subsequent treatment for several cancers (Herbst et al., 2013; Powles et al., 2014). In contrast, chemotherapies pose the risk of resistance mechanisms, destruction of healthy tissue, and unwanted side effects; immunotherapies [e.g., immune checkpoint inhibitors and oncolytic viruses (OVs)] represent attractive alternative therapies that utilize the body’s own immune system to attack cancer cells, thereby leaving healthy tissues/organs unharmed. Indeed, investment in immunotherapies, as a leading treatment modality, is evidenced by over 70 immunotherapy drugs in the clinical pipeline and more than 1,000 clinical trials underway across the United States.

OVs are particularly promising for solid malignancies including breast cancer that are intrinsically resistant to other immunotherapies due to their highly immunosuppressive tumor microenvironment (TME) exhibited by decreased mutational load and neoantigen expression (Galon and Bruni, 2019). Reprogramming of the TME by OVs stimulate antitumor responses with efficacy demonstrated in a number of preclinical and early-phase clinical studies including breast cancer (Andtbacka et al., 2015; Bourgeois-Daigneault et al., 2018; Samson et al., 2018). Even though OVs constitute a wide range of viruses, Herpes simplex type-1 virus (HSV-1) is particularly attractive due to its well-characterized pathogenesis of natural infection and clinically proven antivirals, providing a “safety net” to clinical toxicity. HSV-1 in comparison to HSV-2 has also shown significantly higher levels of danger-associated molecular patterns (DAMPs) attributed to coordinating a CD8^+^ T-cell response (Workenhe et al., 2014) which is thought to be critical for the control of tumor growth (Fridman et al., 2012). HSV1716 is a conditionally replication-competent virus derived from HSV-1 strain 17 that fails to replicate in normal non-cancer cells due to a deletion in the RL1 genes encoding ICP34.5. The first FDA-approved OV for melanoma (Johnson et al., 2015) has, since, demonstrated minimal systemic toxicity in over 100 phase I/II trials for patients with solid malignancies (Mace et al., 2008; Andtbacka et al., 2016; Streby et al., 2017); however, its early promising success has stalled as investigators attempt to reconcile the heterogeneity in the clinical response against both solid and disseminated tumors (Kaufman et al., 2022). Whilst, they are a miracle for some; they fail to work for all patients with overall response rates between 15% and 20% (Macedo et al., 2020).

This heterogeneity not only depends on whether a high enough concentration has been delivered to the target cells [which presents another set of challenges reviewed here (Howard and Muthana, 2020)] but also on a number of factors that influences viral infection; and therefore, OV-mediated antitumor therapeutic responses including; 1) the type of virus used and pre-existing immunity (Chen et al., 2000; Ricca et al., 2018); 2) the type of cancer being targeted (their immune phenotype and genomic mutation profile) (Maleki Vareki, 2018; Bonaventura et al., 2019); and 3) metabolic, nutritional, and microbiome status (Harper et al., 2020; Sumbria et al., 2020). Preclinical modeling of this milieu of interactions is crucial if we are to see OVs reach their full potential, yet immune-oncology modeling is arguably the most challenging problem translational scientist’s face (Bareham et al., 2021).

Preclinical assessments for the therapeutic potential of oncolytic herpesviruses are heavily reliant on immunocompromised mouse models (Speranza et al., 2016). Xenograft models involving immunocompromised mice–bearing human tumor cell lines or whole tissue [patient-derived xenograft (PDX) models] offer high reproducibility and improved preservation of the biological and histopathological features of the original tumors, respectively (Derose et al., 2011). However, the former has demonstrated poor correlation with clinical results (Kerbel, 2003) [particularly subcutaneous models that are not orthotopic (Killion et al., 1998)] due to the differences between human and mouse biology (Mestas and Hughes, 2004), and the latter is costly due to low engraftment success rates and long establishment times. Additionally, lymphocyte-mediated responses to the tumor will be lost when using immunocompromised mice, whereby nude mice lose certain T-cell responses and SCID mice lose both their T- and B-cell responses (Belizário, 2009). To overcome this, humanized PDX models have been utilized to model the efficacy of CAR-T therapies by co-engraftment of a human fetal thymus to mimic a human functional immune system (Mhaidly and Verhoeyen, 2020); however; these are highly complex and expensive.

Syngeneic immunocompetent models allow for low-cost longitudinal study of the paradoxical role of immune cells in both tumor progression and elimination as well as safety and toxicity of OVs, but the mouse strain in both syngeneic and xenograft models will contribute to the immunophenotype and hence response to OV treatment. A summary of immunocompetent models for the study of oncolytic herpesviruses by Speranza et al. (2016) demonstrates the range of responses seen with efficacy predominantly relying on either intratumoral inoculation or combination therapy in comparison with immunocompromised studies. The validation of results in multiple models is often regarded as the best practice but inconsistencies between models as described can hinder the interpretation of clinically relevant data versus technical artifacts. Here, we present a series of conflicting interventional efficacy studies using HSV1716 for the treatment of breast cancer in syngeneic orthotopic mouse models. These immunocompetent models are required to understand the mechanistic biology of OVs, but in an attempt to recapitulate our previous success (Howard et al., 2022), we have uncovered valuable determinants of viral toxicity, and the heterogenic immune responses seen.

## Materials and Methods

### Ethics Statement

Animal procedures were carried out in accordance with the UK Animals (Scientific Procedures) Act 1986 with approval from the UK Home Office approval (PP1099883), the ARRIVE (Animal Research: Reporting of *In Vivo* Experiments) guidelines, and the University of Sheffield Animal Welfare Ethical Review Body (AWERB).

### Cell Lines

Mouse mammary cancer cells EO771 (obtained from Dr. Jessalyin Ubellacker, Harvard University, United States), 4T1-Luc-BR (obtained from Prof. Sanjay Srivastava, University of Texas, United States), and PyMT-TS1 (American Type Culture Collection, ATCC) were cultured in a DMEM growth medium supplemented with 10% v/v fetal bovine serum (FBS, Gibco, Invitrogen, Paisley, United Kingdom), in a humidified incubator under 5% v/v CO_2_ conditions. E0771 and 4T1 cells were stably transfected to express luciferase cultured in DMEM +10% FCS (Gibco, Invitrogen, Paisley, United Kingdom). The identities of all cell lines were regularly confirmed using microsatellite analysis and were tested to be free of mycoplasma.

### Viruses

HSV1716 and GFP expressing HSV1716 were obtained from Virtuu Biologics Ltd. in stocks of 1 × 10^8^ particle-forming units (PFU) in compound sodium lactate (Hartmann’s solution) with 10% v/v glycerol. HSV1716 is derived from HSV strain 17+ with deletions of both copies of the RL1 gene encoding for the neurovirulence factor ICP34.5 (HSV1716). HSV1716-GFP has a green fluorescent protein (GFP) added to the RL1 gene locus and is driven by the phosphoglycerate kinase (PGK) promoter (Conner et al., 2008). All vials were stored at −80°C and freshly thawed on ice in 0.1 ml aliquots immediately before each experiment.

### *In Vivo* Studies

Female C57Bl/6, FVB, or Balb/c mice were obtained from Charles River Laboratory (Kent, United Kingdom) at 6–8 weeks and acclimatized in the Biological Services Laboratory for 7 days prior to experimentation. The animals were maintained on a 12:12 h light/dark cycle with free access to food and water. The animals were anesthetized using 3%–4% v/v isoflurane in 70%:30% v/v N_2_O:O_2_.

### Experimental Design

For tumor growth in female immunocompetent mice (*n* = 3–9/group), 3 × 10^5^ mLUC-E0771, 3 × 10^5^ PyMT-TS1, and 1 × 10^5^ mLUC-4T1 cells were injected into the inguinal mammary fat pads of C57Bl/6, FVB ,and Balb/c mice, respectively, in 50% matrigel: 50% PBS. Mammary tumor growth was assessed by digital caliper measurement every 2–3 days, and when tumors reached ∼100 mm^3^, mice were randomly divided into groups and treated with either PBS or HSV1716 (concentration range 1 × 10^5^–1 × 10^7^ PFU/mouse). Further experimental details pertaining to each model are described as schematics in the appropriate figures. Of note, the animals implanted with luciferase-expressing cell lines were imaged using a luminescence *in vivo* imaging system (IVIS Lumina II imaging, Caliper Life Sciences) following the intraperitoneal injection of D-luciferin (150 mg/kg, Invitrogen). This was to track any metastatic burden. The assessment of the condition of mice following OV administration was attributed to the following health score. A score of five indicated a healthy mouse. A point was deducted for displaying each of the following symptoms: pallor, respiratory distress, piloerection, reduced mobility, and swelling.

### Clinical Chemistry

The systemic toxicity of the virus was assessed in plasma samples using a Roche Cobas 8000 analyzer at the Department of Clinical Chemistry, Royal Hallamshire Hospital, Sheffield Teaching Hospitals NHS Trust. Alanine transaminase (ALT), aspartate transaminase (AST), and alkaline phosphatase (ALP) were measured as increases in the concentrations of these liver function tests indicating liver or muscle damage. We also measured intracellular fluids including potassium, phosphate, and uric acid which are associated with the rapid release and metabolism of intracellular nucleic acids as a marker of tumor lysis syndrome.

### Tissue Analysis

Tissue (tumors, spleen, and liver) was harvested from mice after being killed with half of the tissue being embedded in an OCT freezing medium, and half was snap-frozen for flow cytometry. Immunofluorescence of tumors was carried out on 4-µm tumor cryosections. The sections were blocked with 1% w/v BSA and 5% v/v goat serum for 30 min and incubated, at room temperature, with primary conjugated antibodies against CD3 (1:200 dilution, BD Pharmingen), CD4 (1:50 dilution, BioLegend), CD8 (1:100 dilution, BioLegend), F4/80 (1:100 dilution, BioLegend), and GFP (1:100 dilution). After 1-h, the sections were counterstained with 50 ng/ml DAPI solution and mounted with ProLong™ Antifade (Thermo Fisher Scientific). Images were captured with a Life Technologies EVOS FL Auto at ×20 magnification with DAPI, GFP, RFP, and Cy5 light cubes. Five fields were captured per slide, and the number of positive cells was expressed as an average per field of view.

### Cytokine Bead Array

Serum samples and tumor tissue lysates underwent cytokine bead array (CBA) analysis to assess the expression levels of a series of cytokines. Mouse flex sets were obtained from BD Biosciences and included IL-4, IL-12, IFN-Y, TNF, and GM-CSF. Each BD^TM^ CBA Flex Set contained two vials of standard and one vial each of capture bead and PE detection reagent. The formulization of the capture bead and PE detection reagent components was carried out to a 50× concentration to confirm product performance when multiplexed. An Attune autosampler was used to read the samples.

### Flow Cytometry

In brief, tumors, spleens, and livers were dispersed by enzymatic digestion after first dicing into pieces approximately 1 mm^3^. Tissue pieces were incubated for 30 min at 37°C in serum-free IMDM (VWR International, PA, United States) supplemented with 2 mg/ml dispase, 0.2 mg/ml collagenase IV (Sigma Aldrich, St. Louis, MO, United States), and 100 U/ml DNase (Merck Millipore, Burlington, MA, United States). Dispersed tissues were passed through 70-µm nylon filters (Becton Dickinson, Franklin Lakes, NJ, United States), permeabilized *via* the FOXP3 Fixation/Permeabilization kit (eBioscience), and analyzed for the expression of different markers: pro-inflammatory monocytes (CD14^+^/CD16^+^), immunosuppressive monocytes (CD14^+^/CD163^+^), T_Helper_ (CD3^+^/CD4^+^), T_Reg_ (CD3^+^/CD4^+^/FOXP3^+^), and cytotoxic T cells (CD3^+^/CD8^+^). All antibodies were sourced from BioLegend and used at a concentration of 2 µl per test. The membrane-impermeant, fixable, amine-reactive dye Zombie UV™ Fixable (BioLegend) was used to discriminate between live and dead cells. Flow cytometry was performed using an LSRII flow cytometer (BD Biosciences), and data were analyzed by FlowJo software.

### Human Pleural Effusion Samples

Human samples were obtained from a phase I/IIa trial of intrapleural administration of HSV1716 for the treatment of mesothelioma (NCT01721018). The participants and study design are published in Danson et al. (2020). The samples from patients (*n* = 4) given four doses of HSV1716 were chosen to reflect the multiple dosing performed in the murine studies. The cell populations from pleural effusions were analyzed by flow cytometry using the same markers, as described earlier but with antihuman antibodies (all antibodies were sourced from BioLegend and used at a concentration of 2 µl per test). The cell viability of 2/4 samples was significantly affected by long-term storage; therefore flow cytometry data represent *n* = 2. The following NanoString nCounter™ gene expression analysis was performed with data from two samples described and reported. Amplification-free gene expression profiling of pleural effusions using a NanoString nCounter™ FLEX platform and the nCounter™ PanCancer Immune Profiling Panel, which consist of 750 immune-related genes and 20 housekeeping genes (NanoString Technologies Inc.), was undertaken. For this, total mRNA was extracted using the RNeasy™ Mini Kit (QIAGEN) and quality controlled using a NanoDrop™ 8000 spectrophotometer. For gene expression profiling, 150 ng of total RNA from each sample was used for NanoString probe hybridization which was undertaken overnight (20 h) at 65°C in a PCR machine with a heated lid [each reaction mixture contains 5 µl of RNA solution (150 ng), 8 µl of reporter probe, and 2 µl of capture probe]. After overnight hybridization, excess probes were removed using the NanoString nCounter™ Prep Station and magnetic beads; the hybridized mRNA/probe was immobilized on a streptavidin-coated cartridge. The processed cartridge was subsequently scanned, and raw data were generated at high-resolution (555 fields of view, fov) using a NanoString nCounter™ digital analyzer platform and processed using nSolver™ data analysis software (V.4.0). Imaging quality control (QC), mRNA positive control QC, and normalization QC were checked, and all the samples were in line with the quality parameters of NanoString gene expression assays. Differential expression was performed using the nSolver™ Advanced Analysis Module v.2.0.115. Data normalization was performed using the geNorm algorithm for the selection of the best housekeeping genes.

### Statistical Analysis

Group-wise comparisons were carried out using one-way independent ANOVA with Tukey’s multiple comparison test (unless otherwise stated in the figure legends) by GraphPad Prism software version 9.0. Data are expressed as means ± SD, and statistical significance was defined as *p* ≤ 0.05.

## Results

### Tolerability to Oncolytic Viruses is Dependent on the Strain of the Mouse Model

We have recently demonstrated that magnetization of the oncolytic virus HSV1716 enhances tumor targeting resulting in increased tumor elimination and a 50% survival advantage in a C57/Bl6 model of E0771 TNBC (Howard et al., 2022), as well as the ability to steer magnetic macrophages *via* magnetic resonance imaging (Muthana et al., 2015). Whilst these studies overcome some of the limitations associated with systemic delivery of OVs, we have uncovered some interesting differences in response when repeated in other syngeneic, orthotopic models of triple-negative breast cancer (TNBC). The treatment protocol from Howard et al. (2022) was replicated in a Balb/c mouse model using 4T1-Luc-BR cells (Supplementary Figure S1A). Tumor growth was rapid as detected by IVIS imaging of luciferase-expressing 4T1 cells (Supplementary Figure S1B) and caliper measurements (Supplementary Figure S1C). At a mean tumor volume of 100 mm^3^ (day 7 post-implantation), mice received three doses of HSV1716 (1 × 10^6^ pfu/mouse) by intravenous injection 5 days apart. In stark comparison to C57/Bl6 tumor-bearing mice in our previous study, Balb/c demonstrated significant tolerability issues at identical concentrations of HSV1716. This manifested as subacute (approx. 20 min post-administration) respiratory distress, pallor, and reduced activity, resulting in their cull. The mice which did not reach their severity limit were administered with log lower concentrations of HSV1716, but ultimately their health deteriorated above untreated controls by day 10 (Supplementary Figure S1D). From day 20 post-implantation, the body weight of control mice started to decrease (Supplementary Figure S1E), and upon post-mortem, it was noted that primary tumors had invaded the body cavity, demonstrating the aggressiveness of this model. No metastases were evident by IVIS.

**FIGURE 1:**
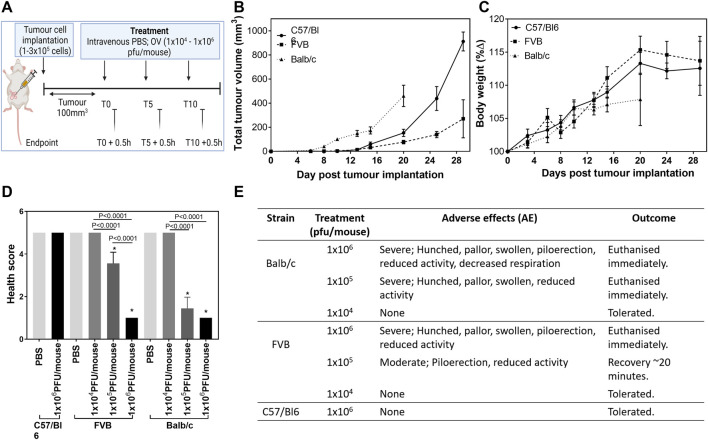
Maximum tolerated dose of HSV1716 is mouse strain-dependent. C57/Bl6, FVB, and Balb/c mice were implanted with mLuc-E0771, PyMT-TS1, and mLuc-4T1-Br breast cancer cells, respectively [**(A)**, created using BioRender], and received a range of OV treatment (1 × 10^4^–1 × 10^6^ pfu/mouse) once average tumor volume had reached 100 mm^3^ to determine the maximum tolerated dose (MTD) **(B)**. Mice receiving their MTD were culled 30 min post-treatment at each timepoint for analysis (*n* = 3/group per timepoint). Health was monitored by measuring body weight **(C)**, and a final health score was calculated **(D)** using severity and duration of adverse effects seen during 30 min observation window **(E)**. Data are shown as mean ± SD. Statistical significance was determined by one-way ANOVA where ∗ = *p*< 0.05 versus PBS group.

Due to the striking difference in response to the virus between the two syngeneic mouse models we undertook a tolerability study using inbred mouse strains well known for their immunological characteristics related to cell-mediated immunity. C57/Bl6 mice (implanted with E0771 cells) and Balb/c mice (implanted with 4T1 cells) display prototypical T-cell subset polarizations with C57/Bl6 mice showing predominant Th1-like immune responses and Balb/c mice predominant Th2 responses (Pinchuk and Filipov, 2008; Radaelli et al., 2018). FVB mice (implanted with PyMT-TS1 cells) represent a balanced profile. A treatment regime of three intravenous injections of HSV1716 at concentrations ranging 1 × 10^4^–1 × 10^6^ pfu/ml was performed once tumors had reached an average volume of 100 mm^3^ (Figure 1A), although due to the difference in tumor growth rate (Figure 1B) the date was determined on a strain by strain basis. Prior to the treatment, body weight measurements suggest that all mice were in comparably good health despite the more aggressive growth of 4T1 tumors (Figure 1C). Following the treatment, the animals were monitored for adverse effects, and cohorts were culled 30 min post-administration as the timepoint at which previous studies succumbed to treatment side effects. E0771-bearing C57/Bl6 mice were unaffected by the highest concentration of virus used in this study (1 × 10^6^ PFU/mouse). This is congruent with our previously published studies (Howard et al., 2022); therefore, lower doses in this mouse strain were not tested. The spleens of all tumor-bearing mice were noticeably larger than those of normal mice, regardless of the strain. In both Balb/c and FVB mice, a linear decline in their health score (Figure 1D) was seen in relation to increasing concentrations of virus (*p* < 0.0001). Adverse events included decreased respiration, pallor, piloerection, and reduced activity (Figure 1E). There was a log difference in the maximum tolerated dose by C57/Bl6, FVB, and Balb/c mice of 1 × 10^6^ pfu/mouse, 1 × 10^5^ pfu/mouse, and 1 × 10^4^ pfu/mouse, respectively.

**FIGURE 2:**
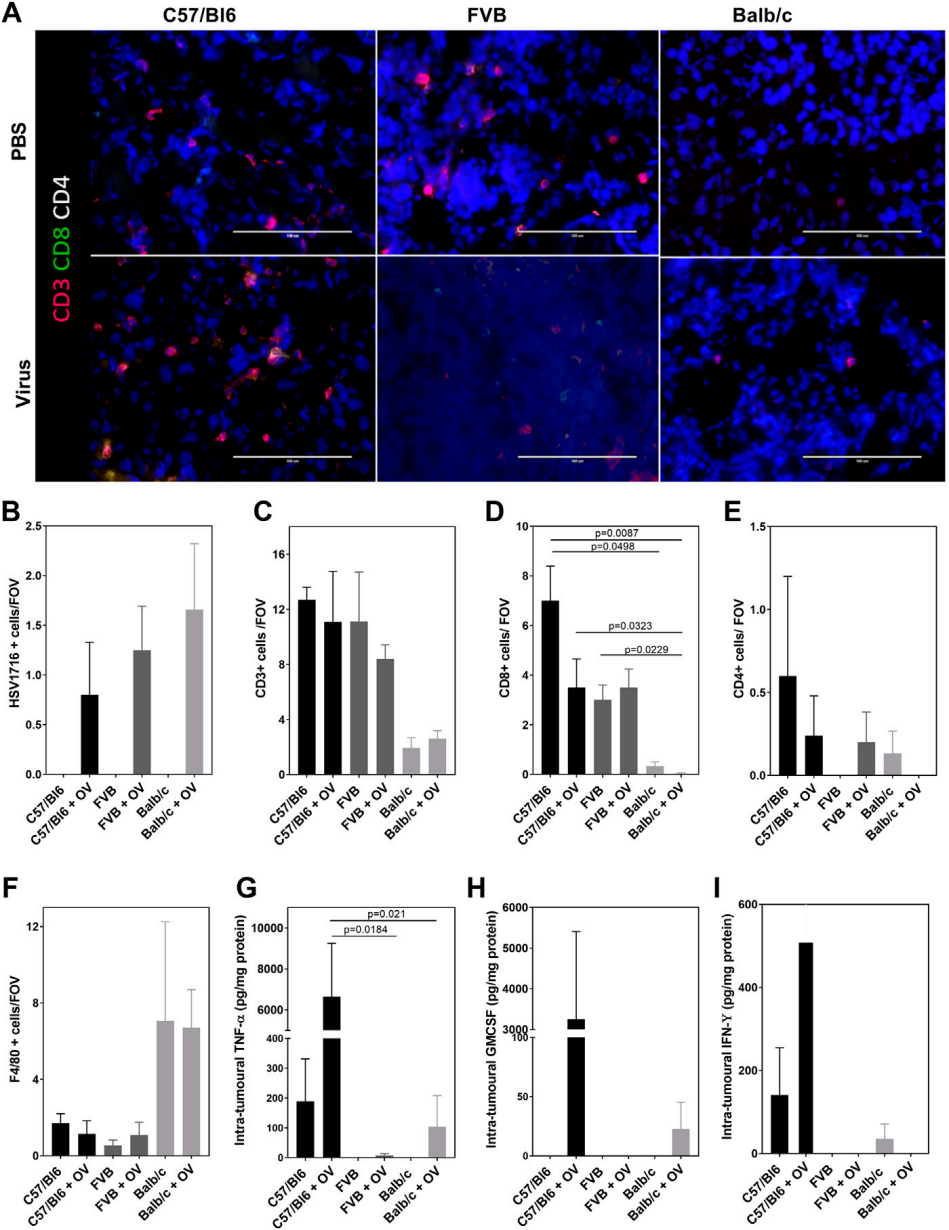
Model dependent T-cell activation. Representative images of tumor sections examined by terminal immunofluorescence staining **(A)** and their quantification of signals for HSV1716 + cells **(B),** CD3^+^ T cells **(C)**, CD8^+^ T cells **(D)**, CD4^+^ T cells **(E)**, and F4/80 + macrophages **(F)**. Intratumoral concentrations of TNF-α **(G)**, GM-CSF **(H)**, and IFN-ϒ **(I)** were detectable using the CBA assay. Data are shown as mean ± SD. Statistical significance was determined by one-way ANOVA with a Tukey *post hoc* test.

The subacute timing of the effects observed together with their anaphylactic-type presentation suggests that this could not be attributed to the preparation of the virus itself but in reaction to the stimulation of immune pathways generating a cytokine storm. A clinical chemistry panel was, therefore, performed to assess the classical biochemical features of lysis of tumor cells. The analysis of plasma was hindered by hemolysis and limited signal detection most likely due to difficulty sampling sick mice and timing of collection (0.5 h post- viral administration), respectively. We attempted to measure tumor lysis syndrome from tumor lysates (Supplementary Figure S2), and whilst data demonstrated differences between the strains of mice (alkaline phosphatase concentration in particular), there was no evidence of cell lysis at this early timepoint despite the presence of HSV1716+ cells in tumor tissue sections (Figures 2A,B). The timing of sampling may be responsible for the lack of changes in the clinical chemistry although this was deemed to be the most appropriate timepoint at which recoverable animals displayed the severest symptoms. Using immunofluorescence, immune populations were characterized to assess the T-helper status within tumors. CD3^+^ T cells were present in tumors of C57/Bl6 and FVB mice in comparison to Balb/c mice (Figures 2A,C). Additionally, these cells displayed a higher proportion of CD8 staining over CD4 (Figures 2A,D,E) with both markers demonstrating a decreasing trend with polarization toward a Th2 phenotype. The pattern of CD8 T-cell activation in tumors of C57/Bl6 mice was substantiated by the presence of intratumoral cytokines known to mediate their effector functions with an increase in GM-CSF, IFN-Y, and TNF-α (Figures 2A,F–H) over other mouse strains. Macrophages were the dominant immune cell present in the tumors of Balb/c mice in the notable absence of T-cell activation (Figures 2A,I).

With the maximum tolerated doses per mouse strain determined, we repeated the efficacy study in PyMT-TS1-bearing FVB mice. A treatment protocol identical to that described in Supplementary Figure S1 was performed (Supplementary Figure S3A), and survival was monitored (Supplementary Figure S3B). Although treatment was tolerated by the mice both immediately post-administration at the reduced concentration and throughout the duration of the study, as determined by body weight (Supplementary Figure S3C), there was no effect on tumor progression (Supplementary Figure S3D) or survival benefits in comparison to PBS-treated controls. This suggests that virotherapy requires a concentration above a threshold dose to elicit an effect (either through saturation of the body with high concentrations or by more targeted delivery).

### Modification of the Immune Phenotype

In an effort to treat our mouse models with concentrations of virus above a threshold dose while avoiding side effects, we attempted to modify the immune environment prior to the administration by pre-treatment with a number of drugs. Vitamin D3 (VD3) is a fat-soluble steroid predominantly known to help maintain the bone health (Chapuy et al., 1992); however, it is also thought to play a role in the adaptive immune system, particularly T-lymphocyte regulation *via* upregulation of Th2 cytokines associated with an anti-inflammatory response (Boonstra et al., 2001). The corticosteroid dexamethasone (Dex) targets inflammation and prevents extension of the cytokine storm, thus preventing the persistence and maintenance of the immune system (Elenkov, 2004). Antihistamines such as diphenhydramine (DPH) are used to inhibit histamine production through alteration to the Th1/Th2 balance in basophils and T cells by increasing the stimulation of Th1 cells and release of IL-2 and IFN-Y while inhibiting Th2 activation (Kato et al., 1999).

**FIGURE 3:**
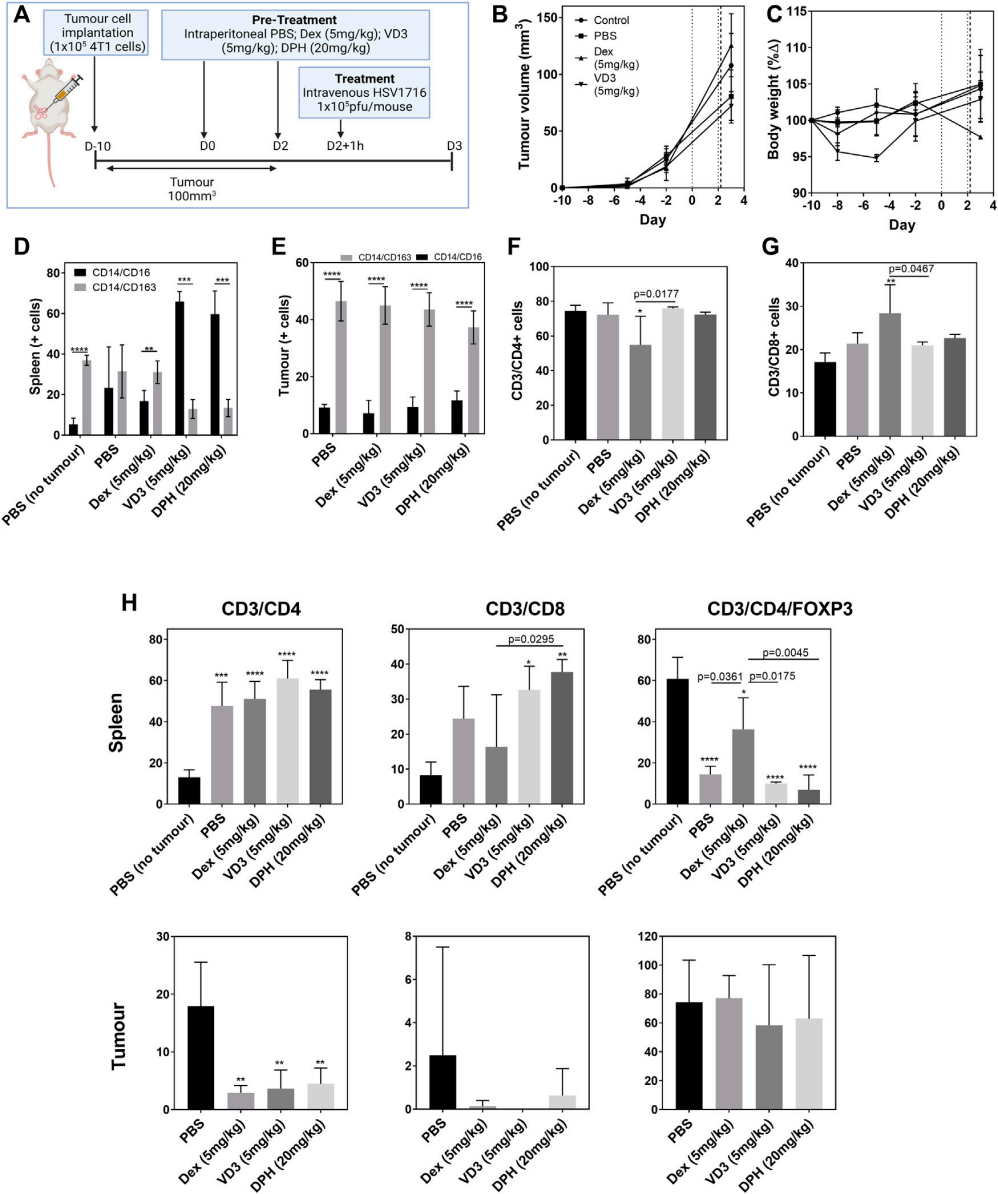
Prophylactic immunomodulation to enhance OV efficacy. Tumor-bearing Balb/c mice (*n* = 4/group) were pre-treated intraperitoneally with different immunomodulators (vertical dotted lines) prior to intravenous OV administration (vertical dashed line) [**(A)**, created using BioRender] in an effort to alter the immune microenvironment for enhanced efficacy and tolerability. Tumor volume **(B)** and body weight **(C)** were measured prior to killing 24 h post OV treatment. Dissociated cell populations from spleen **(D)** and tumor **(E)** samples were analyzed by flow cytometry for pro-inflammatory (CD14^+^/CD16^+^) or immunosuppressive markers (CD14^+^/CD163^+^). Lymphocytes harvested from blood samples were positively selected for CD4^+^ **(F)** and CD8^+^ **(G)** T-cell markers. T-cell analysis from cell populations within spleen and tumor samples was also quantified **(H)**. Data are shown as mean ± SD. Statistical significance was determined by one-way ANOVA with a Tukey *post ho*c test where ∗ = *p* < 0.05, ∗∗ = *p*< 0.001, and ∗∗∗∗ = *p*< 0.0001 versus PBS no tumor.

The Balb/c model was chosen to study the prophylactic modification of the immune profile due to the sensitivity to HSV1716 described earlier. 1 × 10^5^ 4T1 cells were allowed to grow to an average volume of 100 cm^3^ prior to virus treatment. During this time, two doses of PBS, Dex (5 mg/kg), VD3 (5 mg/kg), and DPH (20 mg/kg) were administered intraperitoneally 48 h apart with the second dose 1 h prior to virus treatment (Figures 3A,B). OVs were tolerated at a concentration of 1 × 10^5^ pfu/mouse by all groups, and mice were culled 24 h later in order to evaluate T helper cell activation following treatment. Our previous studies have shown that the cessation of treatment results in tumor regrowth (Howard et al., 2022); therefore, the timepoint was selected to ensure immunological changes following a single virus dose were detected. No adverse effects as a result of treatment with immunomodulators were observed, and body weights remained stable throughout the study (Figure 3C). The changes in clinical chemistry as an evidence of tumor lysis syndrome were undetectable in terminal serum samples (Supplementary Figures S4A–H).

The overall inflammatory status was measured by flow cytometry from dissociated tumor, spleen, and liver cell populations (Figures 3D–F). Within 4T1 tumors, the population of immunosuppressive CD14^+^CD163^+^ cells was 4-fold greater than that of pro-inflammatory CD14^+^CD16^+^ cells (*p* < 0.0001, Figure 3D). Pre-treatment with the immunomodulators did not change these ratios at the tumor level. The analysis of splenocytes indicates a dominant immunosuppressive population in non-tumor–bearing Balb/c mice (*p* < 0.0001, Figure 3E) with dexamethasone also displaying immunosuppressive properties within the spleen as expected. Interestingly, both VD3 and DPH stimulated a pro-inflammatory response with a 5.4-fold increase in CD14^+^CD16^+^ splenocytes (*p* < 0.0001). T-cell populations within these tissues were quantified from viable lymphocytes as the parent population and antibodies were used to select for CD4^+^ T cells (CD3^+^/CD4^+^), CD8^+^ cytotoxic T cells (CD3^+^/CD8^+^), and Tregs (CD3^+^/CD4^+^/FOXP3^+^) (Figure 3G). CD4^+^ T cells were the dominant population within the blood of both tumor-bearing and non-bearing mice treated with PBS (Figure 3H). Treatment with Dex induced a significant decrease in CD4^+^ T cells (*p* < 0.05 vs. PBS no tumor) and a significant concomitant increase in CD8^+^ T cells (*p* < 0.01). The presence of tumors stimulated a large CD4^+^ T-cell response in the spleen when compared to non-tumor–bearing mice (*p* < 0.0001); this was unaltered by the use of the immunomodulators. There was also a trend for increasing the presence of CD8^+^ T cells within the spleen in tumor-bearing mice with the administration of VD3 and DPH also contributing, resulting in a significant increase (Figure 3H). Therefore, 60% of CD4^+^ T cells in spleens of untreated non-tumor–bearing mice expressed FOXP3 as a marker for Tregs (*p* < 0.0001). FOXP3 expression decreased in tumor-bearing mice demonstrating a switch to activate CD4^+^ T cells. Treatment with Dex significantly increased the proportion of Tregs within the spleen compared to PBS-treated tumor-bearing mice (*p* = 0.0361). Within the tumor itself, the majority of CD3^+^ cells were identified as Tregs regardless of immunomodulation. CD4^+^ T cells were more prominent compared to CD8^+^ T cells as we have seen previously with this model (Figure 2). A significant decrease was induced by all immunomodulators (*p* < 0.01) (Figure 3H).

### Human Efficacy is Driven by Th1 Response

**FIGURE 4:**
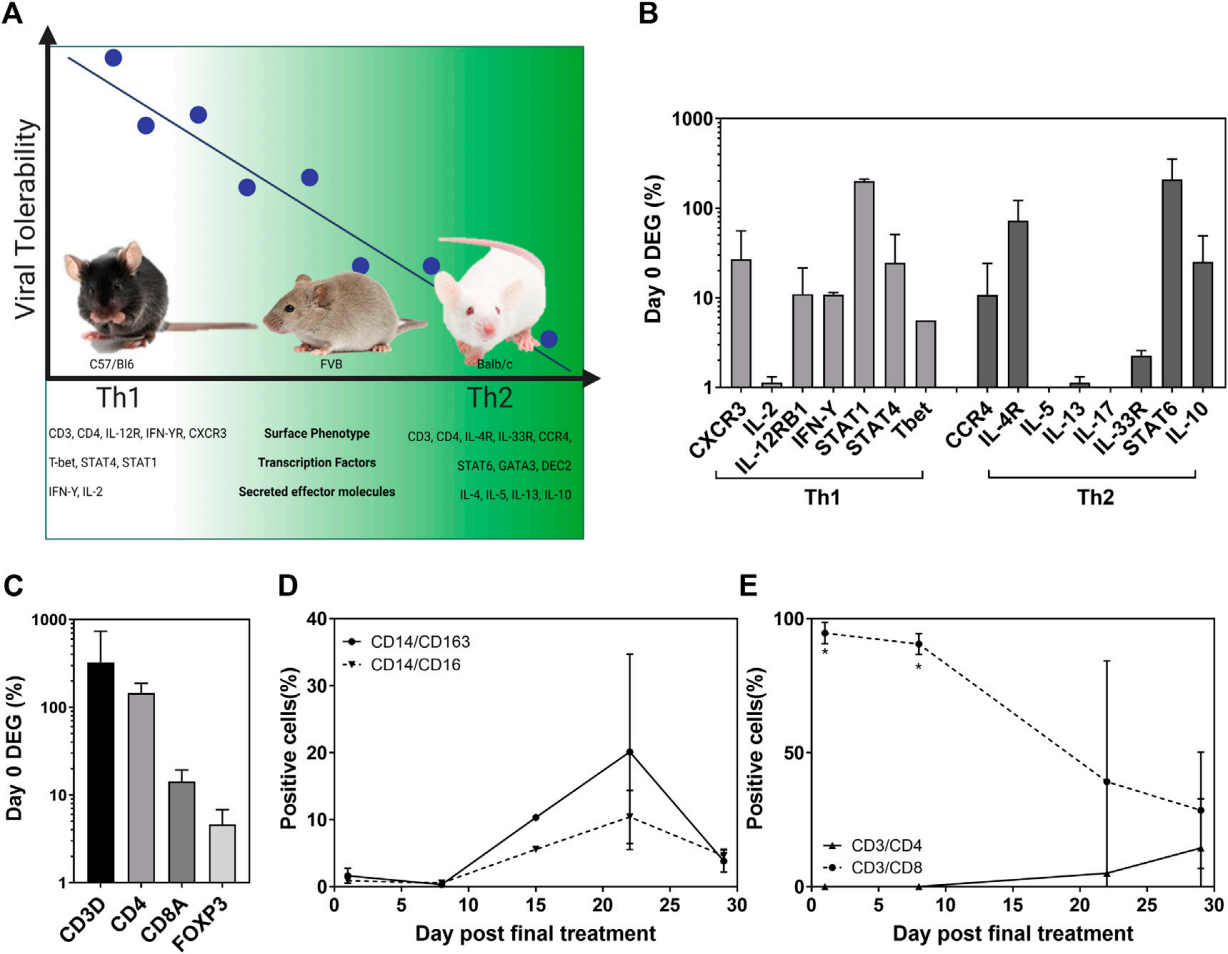
Viral tolerability correlates with host Th bias in both mice and humans. Murine tolerability of HSV1716 correlated with T helper cell polarization associated with prototypical strains of inbred mice [**(A)**, created using BioRender]. Pleural effusion samples taken from MPM patients (*n* = 2), having received four doses of HSV1716, revealed a Th1-dominant cytokine pattern **(B)** and T-cell activation **(C)**, following the analysis of differentially expressed genes (DEG) by NanoString. Flow cytometry of cell populations demonstrated an overall balanced immune system in the weeks following treatment **(D)** with a significant CD8^+^ T-cell–mediated response which declined over time **(E)**. Data are shown as mean ± SD. Statistical significance was determined by Students t-test where ∗ = *p*< 0.001.

Inconsistencies in the efficacy data from the mouse models described make it difficult to define clinically relevant information. However, these “negative” data describe a pattern of effects that correlates with the host’s immunophenotype (Figure 4A). Viral tolerability in our studies correlated with T helper cell polarization displayed by inbred strains of mice. C57/Bl6 mice present with a Th1 dominance allowed the tolerability of concentrations ∼1 × 10^6^ pfu/mouse safely and perhaps even higher. Conversely, 1 × 10^4^ pfu/mouse was determined as the maximum tolerated dose (MTD) by Th2-type Balb/c mice. Interestingly, the MTD of an inbred mouse strain that represents a balanced immunophenotype (FVB) lay between its polarized counterparts at 1 × 10^5^ pfu/mouse. We investigated the clinical relevance of this finding using pleural effusion samples from a phase I/IIa trial of intrapleural administration of HSV1716 which reported good tolerability among patients as well as an antitumor immune response (Danson et al., 2020). Here, we reported a 5–10 fold increase in IFN-ϒ, IL-2, and TNF-α cytokine levels in pleural fluid from 8/11 patients following HSV1716 treatment, representing robust Th1 responses. The differential expression of transcription factors involved in the pathways for cytokine production supports the concentrations of Th1 cytokines measured (Figure 4B). An increase in IL-12 signaling *via* its receptor activates Stat4, which upregulates IFN-Y transcription. IFN-Y proceeds to activate Stat1 which upregulates T-bet, further enhancing IFN-Y production. Although Th2 signaling is mediated by IL-4 receptor activating Stat6 (as seen in Figure 4B), the activation of Stat4, Stat1, and T-bet inhibits GATA3 required for IL-5 and IL-13 production; hence we observed a downregulation of Th2 genes compared to Th1. The differential gene expression of T-cell markers (Figure 4C) showed a higher proportion of CD4^+^ over CD8^+^ cells; and whilst overall a balanced immune response was noted (Figure 4D), flow cytometry analysis of T-cell populations clearly showed a dominant CD8^+^ cell presence immediately following HSV1716 treatment which slowly decreased with time along with a concomitant increase in CD4^+^ cells (Figure 4E). Despite the small sample size, the human data correspond with our C57/Bl6 mouse model that a Th1 immunophenotype confers tolerability to viral treatment. Moreover, a robust CD8^+^ cell response may mediate the antitumor response seen in both these studies.

## Discussion

These studies aimed to investigate the efficacy of HSV1716 as a SACT for triple-negative breast cancer in three different cell lines: E0771, 4T1, and PyMT-TS1. Due to the selective growth of these cells, each required a different inbred mouse strain: C57/Bl6, Balb/c, and FVB mice. We have previously demonstrated *in vitro* efficacy against all 3 cell lines as well as *in vivo* efficacy in E0771 tumor-bearing C57/Bl6 mice (Howard et al., 2022). Here, we observed severe side effects during recapitulation of this experiment in Balb/c and FVB models which limited the concentration of administrable HSV1716, resulting in poor efficacy. We have shown that the adoption of a more targeted approach for systemic delivery of OVs can increase their concentration at the target tumor through magnetic targeting (Howard et al., 2022) and cell delivery (Muthana et al., 2011; Iscaro et al., 2022) for breast and prostate cancers. Further nano-enabled formulations have shielded OVs from immunosurveillance with or without additional ligands for targeted recognition (Iscaro et al., 2019). Through these strategies, saturating concentrations of virus may be avoided and hence improve tolerability. However, in order to study the progression of metastatic breast cancer and the effects of these immunotherapies on immune cells, *in vivo* models representative of the complex interactions between the different cell types are required. Although preclinical studies have generated promising data, many have not translated to humans. Therefore, models (or combination of models) with greater predictive potential are required, together with a willingness to attempt even the most challenging models. Whilst, the two syngeneic mouse models presented here conflicts with our previous success of magnetized-HSV1716 in C57/Bl6 mice; these responses better represent the heterogeneic human population. Therefore, we sought to understand the drivers behind these reactions.

Our comparison of response to HSV1716 by different inbred mouse strains is striking in the log increments of tolerated doses correlating with host T-cell polarization across the strains. This phenomenon is not seen in non-tumor–bearing mice (unpublished data from our laboratory); therefore, the presence of the tumor must play a role in the activation of the immune system in response to the virus. The subacute appearance of adverse effects experienced by the mice supports this theory that the initiation of an antitumor immune response at the tumor site may cause an early release of neoantigens and induction of a cytokine storm. We hypothesized that how the host responds to the cytokine storm will depend on their Th status (Figure 4A) resulting in the stratification of symptoms seen here in the different mouse strains and doses of HSV1716. However, our attempts at validating this theory by clinical chemistry analysis were not statistically significant at either 30 min or 24 h post-treatment, tumor lysis syndrome (TLS) was also fatal in a Balb/c model of plasmacytoma following the intravenous administration of vesicular stomatitis virus (VSV) although progression was much slower with euthanasia 5–8 days post-treatment and evidence of TLS collected at day 4 (Zhang et al., 2016),demonstrating another variable to consider when designing these experiments. Another study investigating the lytic activity of VSV in a syngeneic flank model of lung cancer reported no tolerability issues following both intratumoral and intravenous treatment although, importantly, these were performed in C57/Bl6 mice (Schreiber et al., 2019).

In order to increase the dose of HSV1716 to achieve efficacy, we pre-treated Balb/c mice (i.e., the most susceptible strain) to investigate the alleviation of the tolerability issues. Subsets of patients undergoing immunotherapy have received systemic corticosteroids, vitamin D, and antihistamines either prior to the initiation of immunotherapy or throughout their treatment protocol to manage drug-induced adverse effects (Harmankaya et al., 2011; Grover et al., 2020). Indeed, prophylactic immunomodulation of our tumor-bearing mice was asymptomatic, following intravenous HSV1716. Also, following the prohibitory nature of these side effects on effectively studying HSV1716 treatment in Balb/c models, it is believed that an allergy-type reaction promotes immune evasion and resistance to immunotherapy, suggesting that this treatment is doomed to fail in these models regardless of the concentration achieved. Our data are in accord with this theory, yet rather than negatively selecting these types of models, we need to work with them as a more accurate reflection of the immune heterogeneity within humans. Additionally, while we did not measure efficacy in our immunomodulatory study, recently published articles have demonstrated that cancer patients who took antihistamines during immunotherapy treatment had significantly improved survival (Fritz et al., 2021; Li et al., 2022) and, therefore, may provide a dual purpose in our inflammatory mouse models. In mice, a contrasting impact of corticosteroids on anti-PD1 immunotherapy has been reported (Maxwell et al., 2018), and CTLA-4 blockade restored T-cell numbers exposed to dexamethasone in a model of intracranial glioma (Giles et al., 2018), highlighting the immunosuppressive properties of such immunomodulators. Again, both these studies were performed in C57/Bl6 mice.

Unfortunately, the inoculum used in the immunomodulatory study was not high enough to demonstrate any observable benefits in terms of reduction of the side effects we saw previously. As with all models of infectious organisms, reproducibility can be problematic even if inoculums are prepared from the same stock as calculations are based on titers at the point they were made and frozen. Long-term storage and freeze-thawing can impact the actual titer, but this is not known until after the infection. The lack of expected recoverable symptoms in the PBS group suggests that the titer was less than anticipated; therefore, we could not fully evaluate whether the immunomodulators enhanced tolerability to the virus. However, this study did provide evidence for the therapeutic manipulation of immune subsets to promote a more pro-inflammatory response including the enhancement of antitumor CD8^+^ T cell levels. This is dependent on both the composition of intratumoral immune infiltrates (Fridman et al., 2012) and CD8^+^ T-cell levels in peripheral blood (Workenhe et al., 2014) as we have shown in samples taken from our C57/Bl6 mice and could explain why this strain is preferable for modeling OVs. Importantly, dexamethasone demonstrated a significant increase in peripheral CD8^+^ T cells, which warrants further investigation. The differences in immunological responses of inbred mouse strains allow for the assessment of responses to pathogens. Factors that determine response to OVs include the tumor microenvironment and immune tumor infiltrates, but these studies also suggest that a genetic predisposition toward a particular Th phenotype may also play a role in the systemic response to OVs. These genetically programed biases in Th1 and Th2 immune responses have been shown to modulate atherogenesis (Schulte et al., 2008). Additionally, it has been reported that natural genetic variation in Th cell bias may also precede clinical disease in humans (Olson et al., 2013). A comprehensive review of the literature provides evidence for these Th biases and how they have influenced the outcome of viral infections, including age, ethnicity, and co-morbidities. Altogether, this suggests that predisposition to a particular Th status is measurable and may indicate which patients will tolerate or even respond best to oncolytic viruses. Indeed, we have shown that HSV1716 was well tolerated by MPM patients (Danson et al., 2020) and that cytokine analysis of pleural fluid demonstrated a Th1 response in a phase I/IIa clinical trial. Although administration was *via* an intrapleural catheter and the sample size was small, these findings corroborate our mouse studies that a Th1 bias is associated with both tolerability and enhanced efficacy. This efficacy was driven by a CD8^+^ T cell-mediated response seen here in both human and C57/Bl6 samples and thought to be critical for the control of tumor growth (Fridman et al., 2012). A study of C57BL/6 mice–bearing syngeneic GL-261 gliomas also demonstrated a survival advantage when an HSV virus–expressing mIL-12 initiated a Th1 response and CD8^+^ cell influx compared with the parent virus (Parker et al., 2000). The modification of OVs for the co-expression of immunostimulatory transgenes is one way to skew toward a desired Th response. Here, we used immunomodulatory drugs in an effort to readdress immune homeostasis in prototypical Th2 Balb/c mice. CD8^+^ T cells were noticeably absent in 4T1 tumors of Balb/c mice in comparison to the other strains investigated but following VD3 and DPH administration an increase in these cells within the spleen was observed and may have eventually contributed to the intratumoral immune infiltrates had this particular study continued. Th bias may, therefore, act as a predictive biomarker for both HSV1716 patient tolerability and response. The correlation between Th polarization and tolerability may be used to stratify dose concentrations, while the identification of Th2 dominant hosts may benefit from co-administration with immunomodulators, OVs expressing Th1 transgenes, or an alternative treatment altogether. This is particularly pertinent while current immunotherapies are a miracle for some, not all patients respond and the lack of biomarkers for their identification is costly for both patients and healthcare providers.

It should be noted that while we have utilized three different inbred mouse strains in our investigations, we have only studied their response to HSV1716. If taken alone, it could be argued that FVB and Balb/c models are not appropriate for such efficacy studies or that OVs should be administered only as an intratumoral therapeutic. However, contextualizing HSV1716 response by comparison to other OVs is confounded by a lack of consensus over the experimental design of immunological studies. As stated by Hensel et al. (2019), “variations in experimental variables such as mouse strain, animal physiology, age, gender, drug combinations, time-points, dose, treatment strategies, tumor sub-types, and tumor inoculation methods can create infinite confounders that influence the immune parameters and need to be considered even for a study with a single agent.” Very few studies (if any) confirm OV efficacy in different immunocompetent mouse strains, yet here, these “inconsistencies” build a bigger picture arguably more applicable to heterogenic human populations. Ultimately, embracing these struggles and reporting the spectrum of responses may be the key to improving translational oncolytic virotherapy.

## Data Availability

The original contributions presented in the study are included in the article/Supplementary Material, further inquiries can be directed to the corresponding author.
