# Supplementary material for: Inconsistencies in Modeling the Efficacy of the Oncolytic Virus HSV1716 Reveal Potential Predictive Biomarkers for Tolerability
Source: Front Mol Biosci. 2022 Jun 15;9:889395. doi: 10.3389/fmolb.2022.889395 (PMC9240779; doi:10.3389/fmolb.2022.889395)
Supplement: Supplementary file 1 [file DataSheet1.docx]

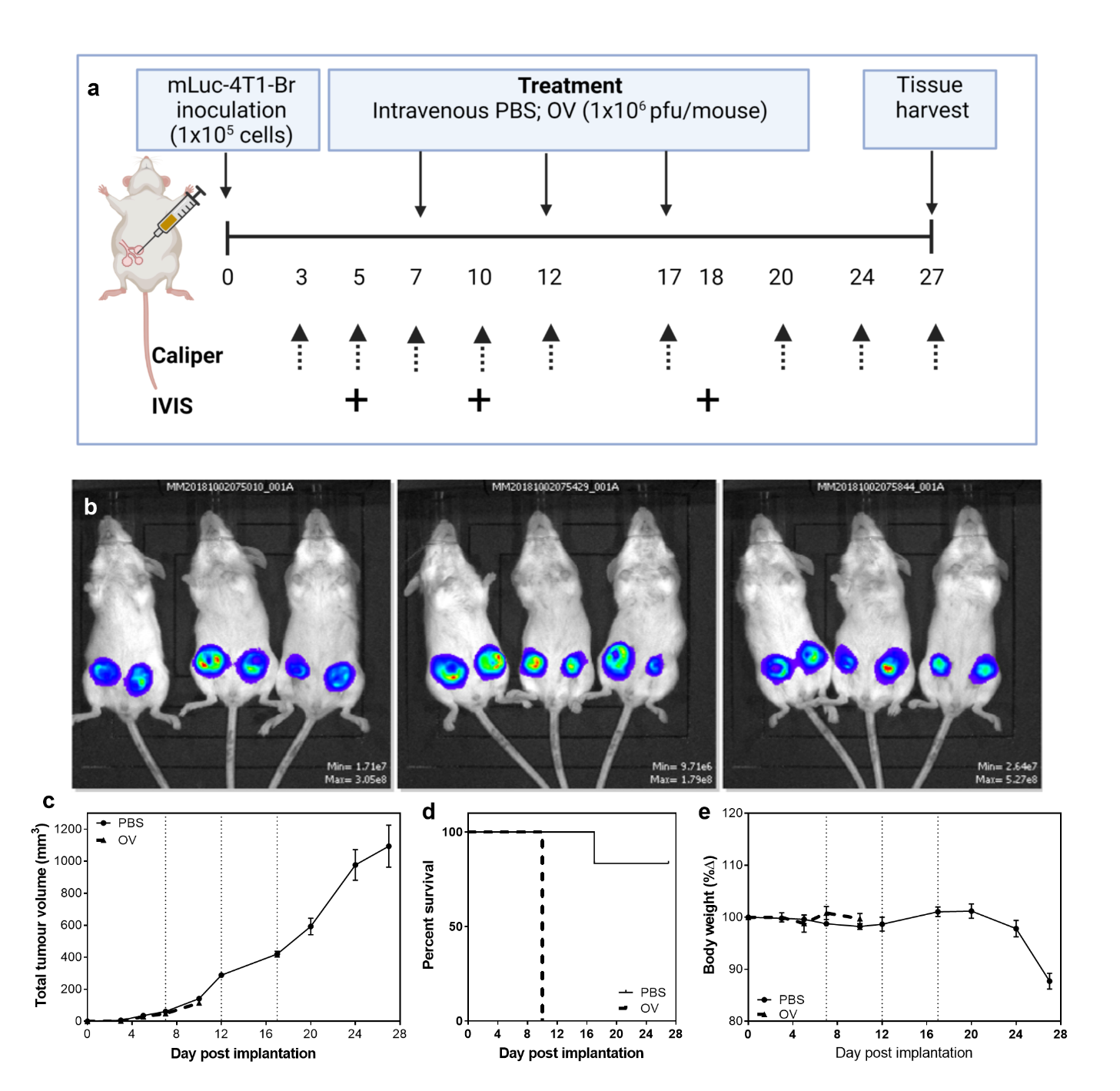


**Figure S1. Tumour bearing Balb/c mice experienced tolerability concerns**. Balb/c mice (n=6/group) were scheduled to receive three intravenous doses (1x10^6^pfu/mouse, vertical dotted lines) of OV (HSV1716) once average tumour size had reached 100mm^3^ (a, created using BioRender). Tumour growth and metastases were visualised by IVIS (b) and volume calculated by caliper measurements (c). Percentage survival (d) and body weight (e) were followed until mice reached their maximum severity limits. Data are shown as mean ± SD.


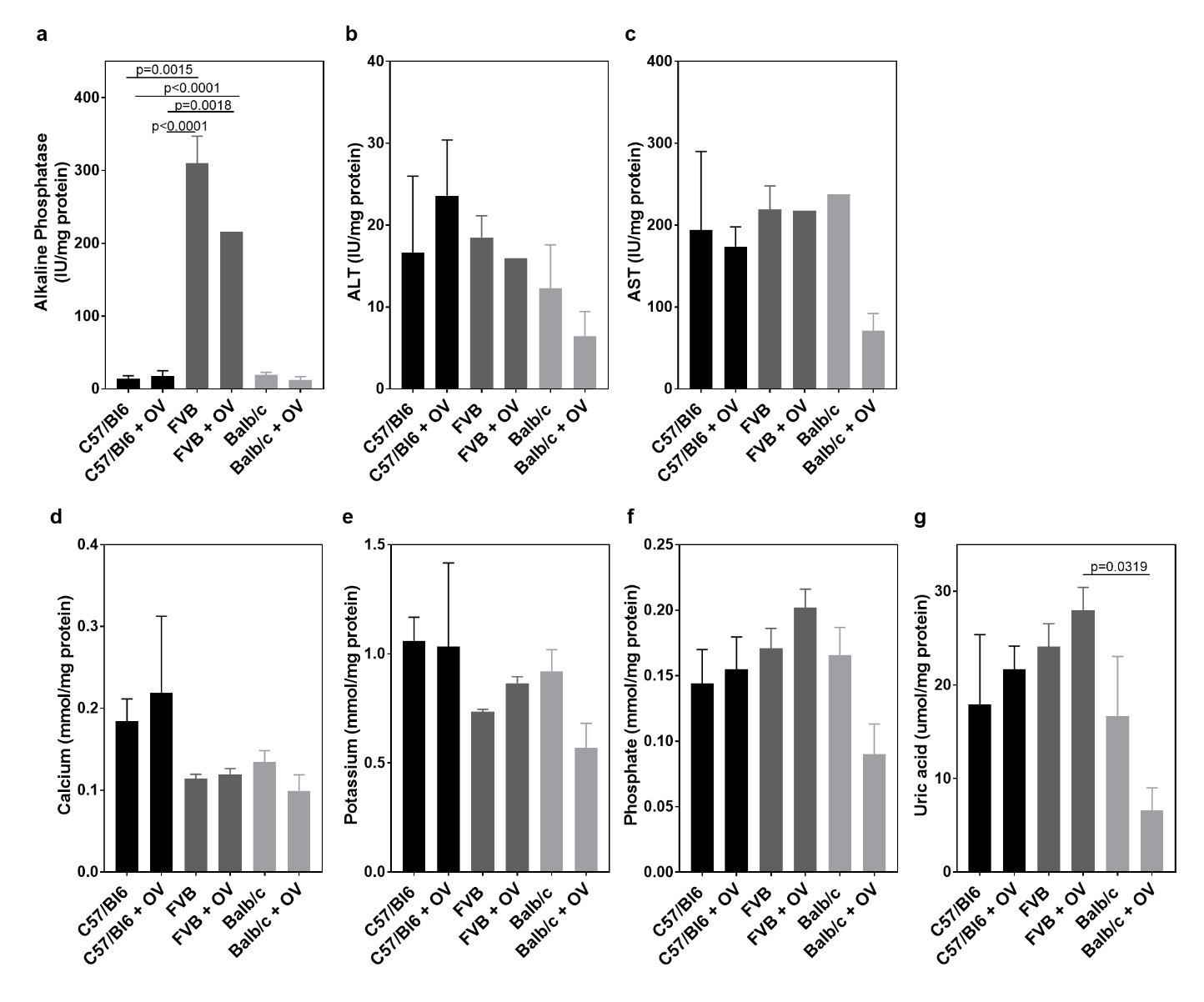


**Figure S2.** Biochemical characterisation of tumour lysates from tumour bearing mice treated IV with PBS or 1x10^6^ pfu/mouse OV. Alkaline phosphatase (a), alanine aminotransferase (ALT, b), aspartate aminotransferase (AST, c), calcium (d), potassium (e), phosphate (f), uric acid (g). Data are shown as mean ± SD. Statistical significance was determined by one-way ANOVA with Tukey’s multiple comparison test.


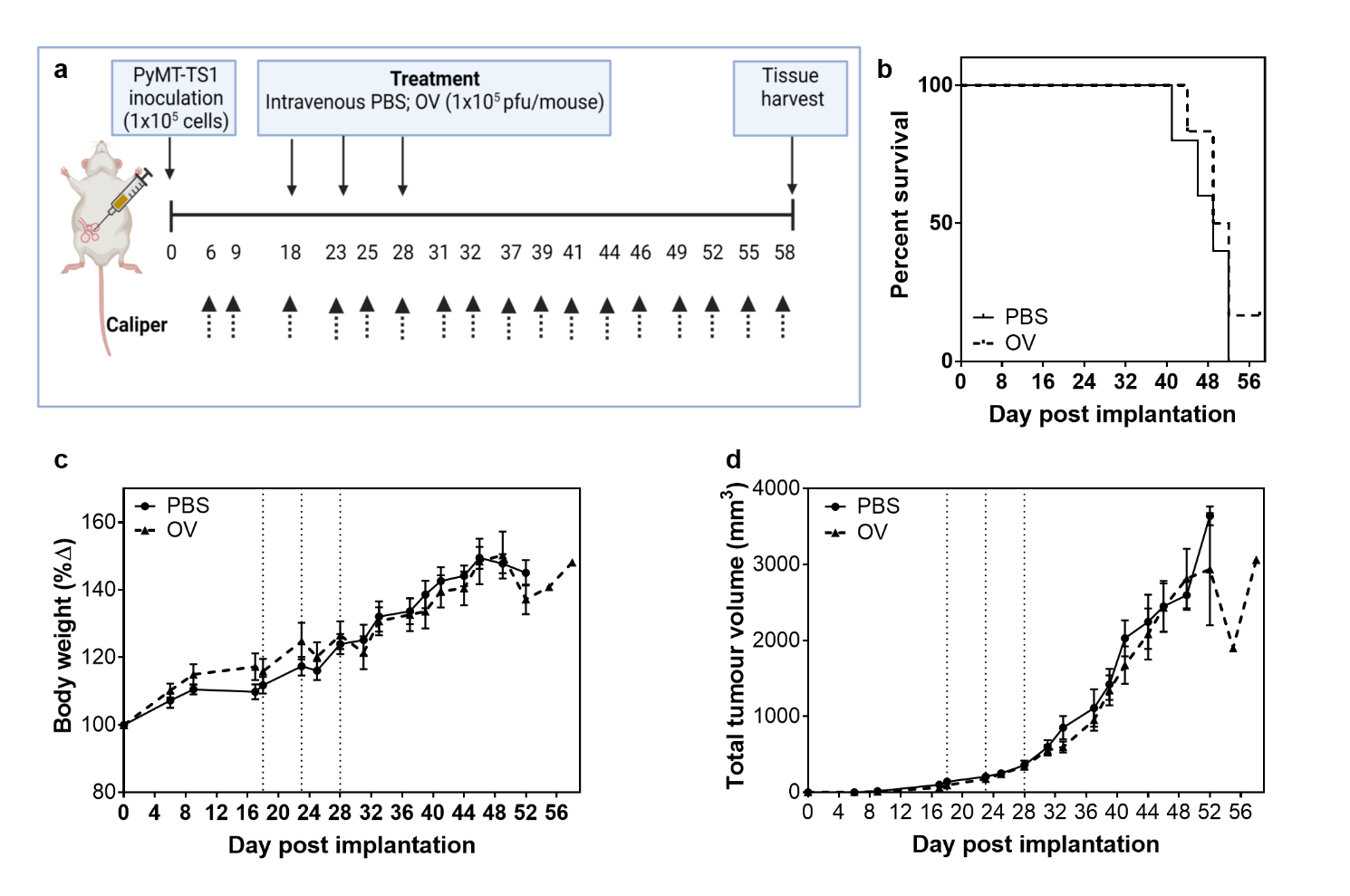


**Figure S3. OV dose limitations affects efficacy**. Tumour bearing FVB mice (n=6/group) received three intravenous doses (1x10^5^pfu/mouse, vertical dotted lines) of OV (HSV1716) once average tumour size had reached 100mm^3^ (a, created using BioRender). Percentage survival (b), body weight (c) and tumour volume (d) were followed until mice reached their maximum severity limits. Data are shown as mean ± SD.


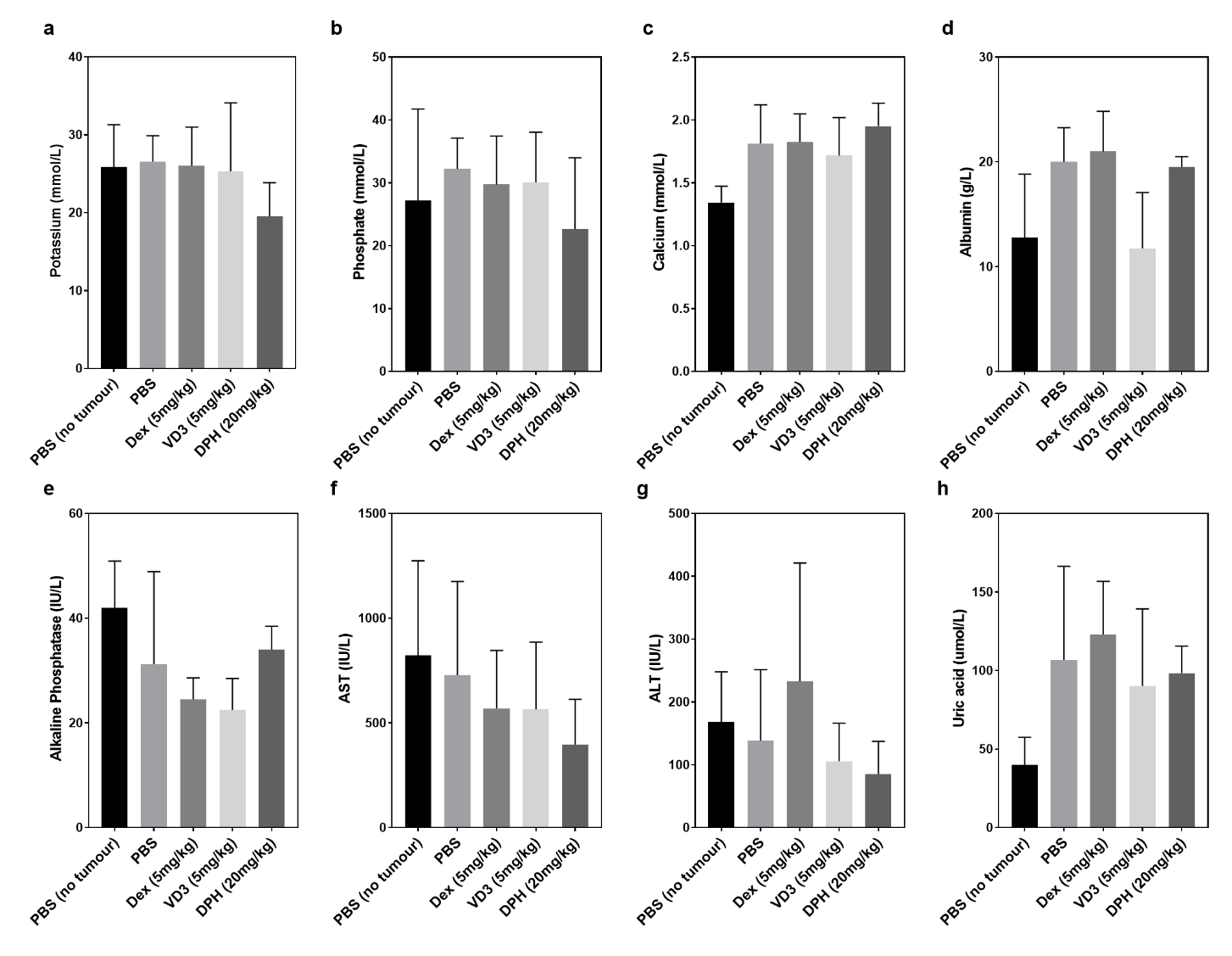


**Figure S4.** Biochemical characterisation of plasma from tumour bearing mice treated IV with PBS or 1x10^6^ pfu/mouse OV following prophylactic immunomodulation. Potassium (a), phosphate (b), calcium (c), albumin (d), alkaline phosphatase (e), aspartate aminotransferase (AST, f), alanine aminotransferase (ALT, g), uric acid (h). Data are shown as mean ± SD.


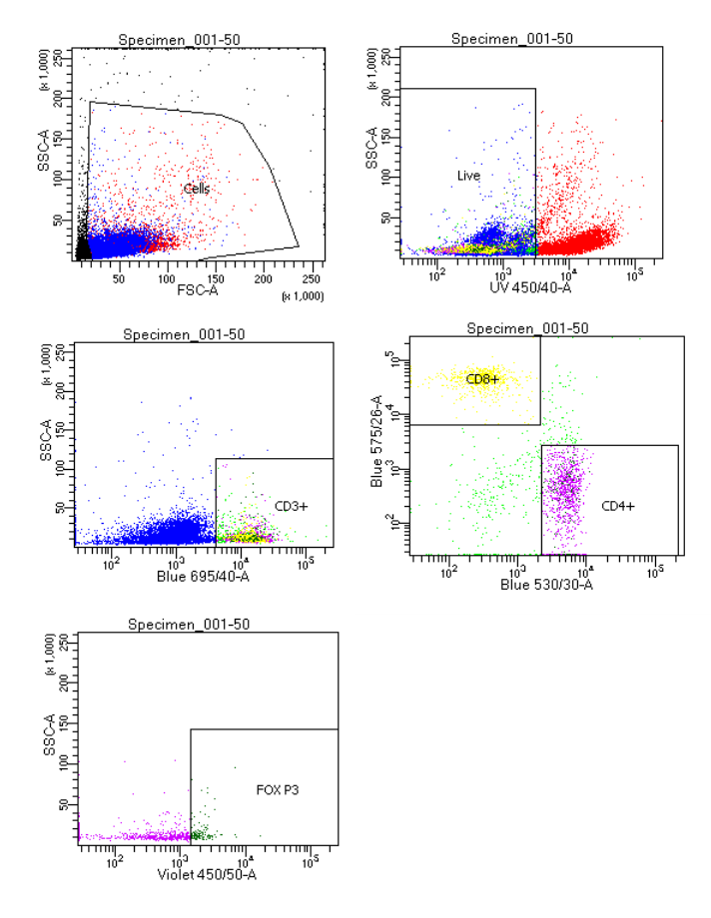


**Figure S5. T cell gating ancestry.** Lymphocyte population were identified by forward scatter vs side scatter. From this, viable cells were identified by the negative staining of a UV viability dye to reduce any autofluorescence from dead cells. T cells were positively selected by their CD3 expression and further analysed for the expression of CD4 and CD8. FOXP3 expression was further selected from CD3/CD4+ parent cells. Spectral overlap was taken into account using compensation beads and unstained, single stained and FMO controls were used to apply the gating strategy.
